# Comparative Analysis of Flavonoids, Carotenoids, and Major Primary Compounds in Site-Specific Yellow-Leaf Tea and Their Dynamic Alterations During Processing

**DOI:** 10.3390/foods14203575

**Published:** 2025-10-21

**Authors:** Jiaqi Yang, Qi Zhou, Shitao Fang, Kangni Yan, Qunhua Peng, Zhi Lin, Haipeng Lv, Dan Mu, Jianyu Fu, Jiang Shi

**Affiliations:** 1 Key Laboratory of Biodiversity Conservation and Characteristic Resource Utilization in Southwest Anhui, Anqing Forestry Technology Innovation Research Institute, School of Life Sciences, Anqing Normal University, North Jixian Road, No. 1318, Anqing 246133, China; 2 Key Laboratory of Biology, Genetics and Breeding of Special Economic Animals and Plants, Ministry of Agriculture and Rural Affairs, Tea Research Institute, Chinese Academy of Agricultural Sciences, Hangzhou 310008, China

**Keywords:** yellow-leaf tea, flavonoids, carotenoids, altitudes, metabolomics

## Abstract

Yellow-leaf tea cultivar ‘Huangjinya’ is valued for distinctive appearance and umami flavor, yet the mechanisms underlying pigment accumulation and flavor formation responding to cultivation and processing remain unclear. Targeted metabolomic quantified 16 carotenoids and 18 flavonoids, revealing significantly higher concentrations in high-altitude site fresh leaves (551.88 ± 7.09 μg·g^−1^ and 213.91 ± 3.78 mg·g^−1^, respectively), approximately 2.8-fold and 1.2-fold higher than in low-altitude site samples. These observed regional differences likely reflect environmental differences between the high- and low-altitude cultivation sites. Flavonoids remained relatively stable during green tea (GT) processing but declined markedly during black tea (BT) production. Carotenoids decreased by 27.91% during green tea (GT) processing but increased by 43.64% in black tea (BT) when using low-altitude site fresh leaves; in contrast, high-altitude site leaves showed a 4.66% increase in GT and a sharp 65% decrease in BT. Thirty-eight primary metabolites showed significant changes responding to altitudes and processing, especially amino acids and oligosaccharides. These findings clarify how altitude and processing affect flavor-related metabolism in ‘Huangjinya’, offering a chemical foundation for improving tea quality.

## 1. Introduction

Tea is one of the most widely consumed non-alcoholic beverages worldwide and well known for its health-promoting properties [1]. The tea plant (*Camellia sinensis*) is remarkably adaptable to diverse environmental conditions, thriving in a variety of climates and altitudes [2]. This adaptability not only influences yield and growth rate but also plays a crucial role in determining tea quality. Among environmental factors, altitude is a key environmental factor associated with the accumulation of key flavor-related metabolites in fresh tea leaves. High-altitude environments often lead to slower plant growth, allowing the accumulation of more complex flavor compounds and contributing to superior taste quality [3,4].

In addition to environmental conditions, postharvest processing is essential in shaping the chemical and sensory attributes of tea [5]. Green and black teas—two of the most consumed types—are produced through distinct manufacturing steps [6]. Green tea processing includes enzyme inactivation (fixation), which preserves catechins and amino acids that contribute to its umami and fresh flavor [7,8]. In contrast, black tea undergoes prolonged withering and fermentation, which promote the enzymatic oxidation of catechins into theaflavins and thearubigins, resulting in a sweet and mellow flavor profile [9]. Furthermore, genetic variations among tea cultivars also influence the accumulation of bioactive compounds such as flavonoids, amino acids, and alkaloids [10,11]. Yellow-leaf tea cultivars, like ‘Huangjinya’, are particularly valued for their light-colored leaves and umami-rich flavor [12]. However, the biochemical basis of these traits, especially how they are influenced by cultivation altitude and processing, is not well understood. Despite increasing interest in yellow-leaf teas, comprehensive studies integrating pigment profiles and flavor metabolites across different altitudes and processing types remain limited.

To address this knowledge gap, the present study investigates both lipid- and water-soluble pigments, along with flavor-related metabolites, in ‘Huangjinya’ tea cultivated at low (~80 m) and high (~600 m) altitude sites. The fresh leaves were processed into both green and black teas, and targeted metabolomics was applied to evaluate the transformations in carotenoids, flavonoids, amino acids, and primary metabolites. The goal was to provide mechanistic insights into how ecological and processing factors influence the formation of tea quality, thereby supporting optimized strategies for cultivar-specific tea production.

## 2. Materials and Methods

### 2.1. Chemicals and Instruments

Methanol (MeOH), acetonitrile (ACN), and ethanol were all MS grade and purchased from Merck (Darmstadt, Germany). Milli-Q water (Millipore Co., Bradford, MA, USA) was used in all experiments. Flavonoid standards (purity HPLC > 98%), including epigallocatechin gallate (EGCG), epicatechin gallate (ECG), epigallocatechin (EGC), epicatechin (EC), gallocatechin (GC), catechin gallate (CG), gallocatechin gallate (GCG), catechin (C), myricetin (M), myricetin 3-galactoside (M-3-gal), myricetin 3′-glucoside (M-3′-glu), quercetin (Q), quercetin 3-galactoside (Q-3-gal), quercetin 3-glucoside (Q-3-glu), kaempferol (K), kaempferol 3-rutinoside (K-3-rut), and myricetin (M), were purchased from Olchemim Ltd. (Olomouc, Czech Republic). The flavonoid stock solutions were prepared at a concentration of 5 mg/mL.

Twenty amino acids (AAs) (purity > 98%), including 19 essential AAs (phenylalanine, leucine, tryptophan, isoleucine, methionine, valine, proline, tyrosine, cysteine, alanine, threonine, glycine, glutamine, serine, glutamic acid, aspartic acid, histidine, arginine, and lysine) and *L*-theanine, were all purchased from Sigma Co. (St. Louis, MO, USA). AA standard stock was prepared at a concentration of 2 mg/mL. Carotenoids were all purchased from Sigma Co. (St. Louis, MO, USA) (purity > 98%), including 3 carotenes (α-carotene, β-carotene, and (E/Z)-Phytoene) and 15 xanthothins (lutein, lutein palmitate, lutein myristate, lutein dilaurate, lutein dimyristate, zeaxanthin, violaxanthin, violaxanthin dibutyrate, neoxanthin, β-cryptoxanthin, 8′-apo-β-carotenal, canthaxanthin, echinenone, β-citraurin, and α-cryptoxanthin), and standard stock was prepared at a concentration of 1 mg/mL. All stock solutions were stored at −20 °C until used.

### 2.2. Sample Collection and Tea Processing

Fresh leaves of the ‘Huangjinya’ cultivar were harvested in March 2024 from two sites in Shengzhou, Shaoxing City, Zhejiang Province, China: a low-altitude site tea garden (~80 m, L_FL) and a high-altitude garden (~600 m, H_FL), both located at 120°49′ E, 29°35′ N. Each sample comprised one bud and two leaves collected from at least 15 independent plants, combined to form a biological replicate. A total of three independent biological replicates were established at each location. After collecting fresh leaves from both high and low altitude sites, we processed them separately into green tea and black tea. (1) Green Tea Processing: ① Wilting (GW): Simply put, spread fresh leaf samples on a withering tray (6–10 cm thick) and wilt them for 4–6 h at 18–22 °C and 58% relative humidity. Leaf moisture is monitored every 5 min using a near-infrared moisture analyzer (MA37-1) from Sartorius (Gottigen, Germany). When moisture reaches 70–72%, the leaves proceed to the next stage. ② Fixation Process: A two-stage fuzzy temperature control technique regulates the high-temperature environment to deactivate enzyme activity in the fresh leaves. Specifically: drum temperature 280–320 °C, hot air temperature 100–110 °C, drum fixation time 2–3 min, drum rotation speed set to 26 rpm, and leaf flow rate 200 kg/hour. Through a feedback mechanism, fixation degree is adjusted based on the criteria of ‘thorough and uniform fixation, disappearance of grassy odor’; leaf moisture content is set to 60–63%. ③ Rolling Process: Place the kill-green tea leaves into a rolling machine (Model 6CR-55) from Fengkai Co (Hangzhou, China) for 45 min of rolling. Program settings: 12 min of no-pressure rolling, 15 min of light-pressure rolling, 12 min of medium-pressure rolling, and 6 min of light-pressure rolling. ④ Drying Process: Post-rolling tea leaves are dried at 130 °C for 15 min until moisture content drops to 20 g per 100 g of tea. Subsequently, spread the leaves for 30 min of initial drying. Finally, place them in a 110 °C hot air dryer for 20 min of secondary drying to ensure complete drying. The finished green tea (GT) is obtained after the drying process. Green Tea Sample Preparation: Collect tea samples (GW and GT) during withering and drying processes. Immediately freeze in liquid nitrogen, freeze dry, and store at −80 °C for later use. (2) Black Tea Processing: ① Withering Stage (BW): Fresh leaves are withered for 12–16 h on an intelligent withering rack (6–10 cm thickness) at an ambient temperature of 18–22 °C and relative humidity of 58%. Leaf moisture is monitored every 5 min using a near-infrared moisture analyzer (NI-MDI). When moisture reaches 58–62%, tea leaves proceed to the next stage. ② Rolling Process (BR): Withered leaves undergo rolling in a rolling machine (Model 6CR-55) for 90 min following this sequence: 25 min of no-pressure rolling → 35 min of light-pressure rolling → 25 min of medium-pressure rolling → 15 min of light-pressure rolling. ③ Fermentation Process (BF): The rolled tea leaves are conveyed to the fermentation chamber, where real-time observation of leaf color changes (from orange-yellow to dark brown) is possible; the system automatically performs online monitoring of temperature and oxygen content to determine when optimal fermentation conditions are reached. ④ Black Tea Production (BT): Fermented leaves are dried at 100 °C for 30 min until moisture content <25%, followed by 30 min of cooling, and subsequently undergo secondary drying in a hot air dryer: dried at 75 °C for 90 min until completely dry. Tea samples are collected at each stage (BW, BF, and BT); immediately frozen in liquid nitrogen; vacuum-dried; freeze-dried; and stored at −80 °C for future use. Both low- and high-altitude site leaves were processed using identical protocols and designated accordingly: L_FL, L_GW, L_GT, L_BW, L_BF, and L_BT for low-altitude and H_FL, H_GW, H_GT, H_BW, H_BF, and H_BT for the high-altitude site.

### 2.3. Targeted Metabolomics of Pigments

Adapting our previous methodologies [13], carotenoids were quantitatively analyzed with slightly modification. Fifty milligrams of tea powder were extracted and purified. The carotenoid contents were detected by MetWare Co. (Wuhan, China) based on the AB Sciex QTRAP 6500 LC-MS/MS platform. Quantitative calibration of 16 identified carotenoids in teas with their equation is listed in the Supplementary Materials.

The absolute quantification of flava-3-ol and major flavonol glycosides was carried out using an ultra-high performance liquid chromatography-quadrupole/electrostatic field orbitrap mass spectrometer system (UHPLC-Q-Exactive/MS; Thermo Fisher Scientific, Waltham, MA, USA). An external standard method was applied for accurate quantitative analysis. The contents of eight flava-3-ols (ECG, EGC, EGCG, EC, CG, GC, GCG, and C) and eight flavonols and glycosides (M-3-gal, M-3′-glu, M, Q, Q-3-gal, Q-3-gluc, K-3-rut, and K) were determined using the external standard method by a preparation of mixture standard working solutions. A stock solution containing standards (5 mg/mL) was diluted to investigate the limit of detection (LOD), limit of quantification (LOQ), and linear ranges of the standards (*n* = 3). A QC sample was used to evaluate the intraday and inter-day precision (*n* = 6).

### 2.4. Widely Targeted Metabolomics Analysis

Nineteen essential amino acids (AAs) and *L*-theanine and primary metabolites (PMs) were analyzed according to our previous methods [14], with slight modifications. Fifty milligrams of lyophilized samples were finely ground and extracted for further MS-based analysis using an Agilent 1290 infinity system (Agilent Technologies, Santa Clara, CA, USA) equipped with a sample manager coupled to a mass spectrometer with an electrospray ion source in both positive and negative ion modes. AAs were quantitatively analyzed by standard verification and calculated using standard curves. PMs were identified according to a self-constructed database including amino acid derivates, alkaloids, organic acids, vitamins, and sugars, according to our previous methods.

### 2.5. Multivariate Statistical Analysis

All analyses were performed in triplicate, and results are expressed as the mean ± standard deviation (SD). Metabolomic data were analyzed using MetaboAnalyst 6.0 (https://www.metaboanalyst.ca). Principal component analysis (PCA) and hierarchical clustering (HCA) were performed, and data visualizations were generated as heatmaps with dendrograms. Metabolites with significant differences were identified using orthogonal partial least squares-discriminant analysis (OPLS-DA), based on variable importance in projection (VIP > 1) and |log2 fold change| > 1. All datasets were log2-transformed and mean-centered prior to OPLS-DA. The validity and predictive ability of the OPLS-DA models were assessed using the R^2^Y and Q^2^Y parameters. The models were further validated against overfitting by conducting a permutation test with 200 iterations. Furthermore, Pearson correlation analyses were performed using the data from the three independent biological replicates (*n* = 3) for each tea product type (e.g., L_GT, H_GT, etc.).

## 3. Results and Discussions

### 3.1. Major Flavonoids Distribution in ‘Huangjinya’ FLs in Different Altitudes

The absolute concentrations of 33 pigments were determined, including 16 flavonoids and 18 carotenoids (Table 1). The total pigments of H_FL (765.79 ± 6.67 mg·g^−1^ DW) were significantly higher than L_FL (372.27 ± 2.83 mg·g^−1^ DW). Among the catechins, EGCG and EGC were the most abundant in both samples. In H_FL, EGCG accounted for 34% (73.39 ± 1.29 mg·g^−1^), followed by EGC at 32% (70.20 ± 1.65 mg·g^−1^). In contrast, L_FL contained 60.92 ± 0.99 mg·g^−1^ EGCG and only 27.72 ± 0.67 mg·g^−1^ EGC, indicating a substantial decrease in EGC at the lower altitude (Table 1).

No significant difference was observed in EC content between the two altitudes. Typically, low-altitude site regions receive more sunlight and higher temperatures, promoting catechin accumulation [15,16]. However, in this case, the catechin concentrations were comparable across altitudes, likely due to the inherent characteristics of the ‘Huangjinya’ cultivar, which is known for a naturally low catechin content [17,18]. Interestingly, the ratios of EGCG/EGC and ECG/EC suggested that the degree of catechin galloylation was inversely related to cultivation altitude. This trend may be attributed to the activity of epicatechin gallate transferase (ECGT), which catalyzes catechin galloylation and exhibits optimal activity at ~30 °C. Since temperature decreases with elevation, reduced galloylation in H_FL could explain the observed lower levels of gallate-type catechins and the milder astringency of high-altitude site tea infusions [19,20].

In addition, the total flavonol glycosides was significantly affected by the altitudes according to the results, with higher flavonol glycosides in H_FLs (7.78 mg·g^−1^) than in L_FL (5.67 mg·g^−1^). M-3-gal occupied the highest content, which reached 3.69 and 4.87 mg·g^−1^ in L_FL and H_FL, respectively, followed by Q-3-gal, which exhibited the opposite tendency of 1.21 mg·g^−1^ in L_FL and 0.90 mg·g^−1^ in H_FL. K showed the lowest level with <0.01 mg·g^−1^. The elevated accumulation of flavonol glycosides in H_FL may be a photoprotective response to higher UV radiation at high altitudes [21,22]. Glycosylation of flavonoids is a well-documented defense mechanism in tea plants under abiotic stress [23,24]. Additionally, increased glycosylation has been linked to enhanced sweetness and aftertaste, supporting previous findings on the sensory benefits of high-altitude site teas [4].

### 3.2. Major Carotenoids Distribution in ‘Huangjinya’ FLs from Different Altitudes

Three carotenes and fifteen xanthophylls were quantitatively analyzed, exhibiting a significant difference (*p* < 0.001) in L_FLs and H_FLs. The total content of carotenoids was found in significantly higher concentrations in samples from the high-altitude site, with a value of 197.02 ± 2.53 μg·g^−1^ in L_FLs and 551.88 ± 7.09 μg·g^−1^ in H_FLs. Lutein was the most abundant carotenoid in both samples, accounting for over 80% of the total. In H_FL, lutein reached 477.74 ± 6.14 μg·g^−1^ compared to 166.35 ± 2.14 μg·g^−1^ in L_FL. This represents a 2.87-fold increase in lutein content in the high-altitude site samples. β-carotene was the second most prevalent, with concentrations of 36.72 ± 0.47 μg·g^−1^ (H_FL) and 12.97 ± 0.17 μg·g^−1^ (L_FL). Zeaxanthin followed at 17.58 ± 0.23 μg·g^−1^ in H_FL and 9.31 ± 0.12 μg·g^−1^ in L_FL.

Carotenoids are essential lipid-soluble pigments in tea leaves, serving as light-harvesting antennae for chlorophyll and as precursors of volatile aroma compounds [25,26]. Their accumulation is highly sensitive to ecological factors, particularly light and temperature. Previous studies suggest that carotenoid biosynthesis is more responsive to temperature changes than light intensity or altitude alone [27]. This marked increase in carotenoid content at the high-altitude site is consistent with the previous literature showing that lower temperatures can induce carotenoid biosynthetic enzymes. For example, carotenoid cleavage dioxygenase 4 (CCD4) has been shown to promote carotenoid accumulation as a mechanism for environmental adaptation [28]. While further transcriptomic or enzymatic assays would be required to confirm this in ‘Huangjinya’, it presents a plausible hypothesis for the observed metabolic shift.

### 3.3. Amino Acids Distribution in ‘Huangjinya’ FLs from Different Altitudes

Free amino acid (AA) content was strongly influenced by cultivation altitude (Table 2). The total concentration of free AAs was significantly higher in the high-altitude site fresh leaves (H_FL: 42.63 ± 0.72 mg·g^−1^ DW) compared to low-altitude site leaves (L_FL: 32.44 ± 0.55 mg·g^−1^ DW). As the most abundant free AA, theanine accounted for more than 60% (19.62 ± 0.33 and 25.96 ± 0.44 mg·g^−1^ in L_FLs and H_FLs, respectively), followed by glutamic acid (3.21 ± 0.05 and 4.03 ± 0.083 mg·g^−1^, respectively), glutamine (2.47 ± 0.04 and 4.02 ± 0.07 mg·g^−1^, respectively), arginine (1.44 ± 0.02 and 2.57 ± 0.04 mg·g^−1^, respectively), and aspartic acid, which mainly contributes to the umami taste in tea, with its content in L_FL (1.99 ± 0.03 mg·g^−1^) being slightly higher than that in H_FL (1.70 ± 0.03 mg·g^−1^).

Free amino acids, particularly theanine, glutamic acid, and glutamine, are known to contribute significantly to the umami and sweet taste of green tea infusions [29]. The elevation-driven increase in these compounds aligns with previous findings suggesting that high-altitude site conditions—characterized by cooler temperatures and reduced solar radiation—suppress carbon metabolism while enhancing nitrogen metabolism in tea plants [30]. These conditions may promote amino acid biosynthesis or reduce their degradation, explaining the higher AA levels observed in high-altitude site leaves. This biochemical adaptation contributes to the superior taste quality often associated with high-mountain teas [31].

### 3.4. Primary Metabolite Profiling in ‘Huangjinya’ Fresh Leaves from Different Altitudes

**Figure 1:**
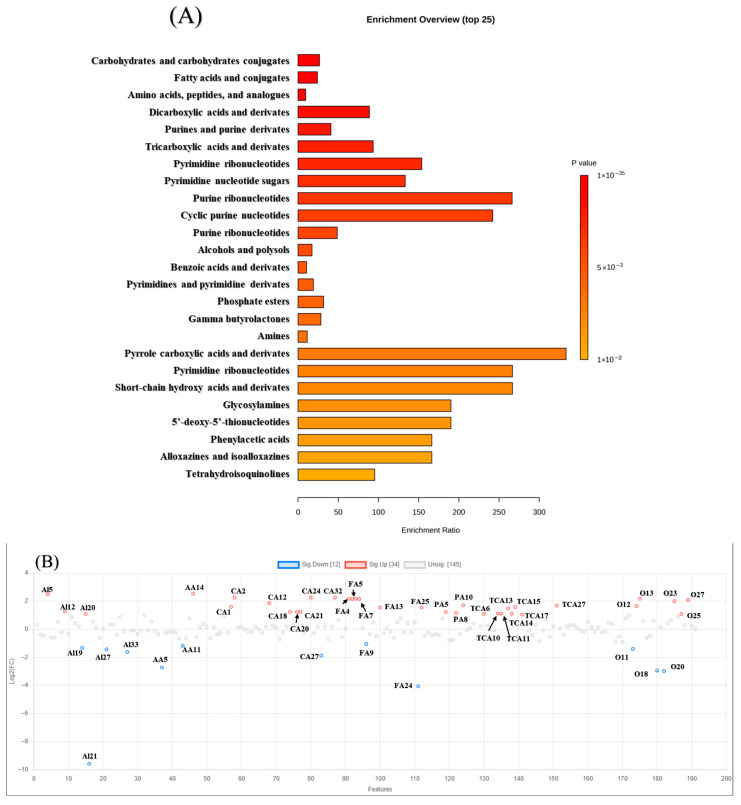

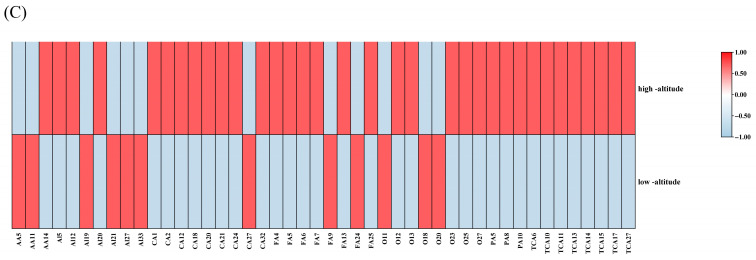
Multivariate analysis of primary metabolites (PMs) and significant differential PMs screening in ‘Huangjinya’ fresh leaves cultivated at different altitudes. (**A**) Pathways enrichments of all identified PMs in ‘Huangjinya’ fresh leaves harvested from two altitudes; (**B**) significant differential accumulated PMs (SPMs) analysis of high- and low-altitude cultivated ‘Huangjinya’ fresh leaves; (**C**) high- and low-altitude site-cultivated ‘Huangjinya’ fresh leaves: peak areas of SPMs (red indicates highest peak area at high elevation; blue indicates highest peak area at low elevation). Note: In total, 201 PMs were identified and named with IDs for further multivariate analysis, as shown in Table S1. AA 5, 11, and 14 were 4-guanidinobutyric acid, glutathione, and *L*-methionine, respectively; Al 5, 12, 19, 20, 21, 27, and 33 were adenosine 2′,3′-cyclic monophosphate, cytidine, guanosine 5′-diphospho-D-mannose, guanosine 5′-monophosphate, histamine, *S*-(5′-adenosyl)-*L*-homocysteine, and uridine 5′-diphosphogalactose, respectively; CA 1, 2, 12, 18, 20, 21, 24, 27, and 32 were 5′-deoxy-5′-(methylthio)adenosine, adonitol, *D*-(*+*)-xylose, *D*-mannitol, *D*-sorbitol, dulcitol, *L*-(-)-arabitol, methyl-*D*-galactopyranoside, and xylitol; FA 4, 5, 6, 7, 9, 13, 24, and 25 were 2-phospho-*D*-glycerate, 3-phospho-*D*-glycerate, 3-phosphoglyceric acid, 4-methy-2-oxo-pentanoic acid, *D*-erythro-dihydrosphingosine, glycerophosphate, sebacic acid, and sn-glycerol 3-phosphate, respectively; PA 5, 8, and 10 were 4-hydroxyphenylacetic acid, homogentisic acid, and *p*-coumaric acid, respectively; TCA 6, 10, 11, 13, 14, 15, 17, and 27 were beta-*D*-glucose 6-phosphate, *D*-fructose 6-phosphate, *D*-galactose 1-phosphate, *D*-glucose 1-phosphate, *D*-glucose 6-phosphate, *D*-mannose 6-phosphate, fructose-6-phosphate, and phosphoenolpyruvic acid.

A total of 202 primary metabolites (PMs) were identified using a self-constructed database, including amino acid derivatives, alkaloids, carbohydrates, tricarboxylic acid (TCA) cycle intermediates, fatty acids, phenolic acids, vitamins, and sugars, which were enriched in crucial pathways (Figure 1A). The validity and predictive ability of the OPLS-DA models were assessed using the R^2^Y and Q^2^Y parameters based on all identified PMs. Among them, 46 showed significant differential accumulation (fold change > 2, *p* < 0.05) between high- and low-altitude site fresh leaves (Table S1; Figure 1B). The detailed changes are illustrated in Figure 1C, according to the peak areas of comparison between the two altitudes samples. Four alkaloids (i.e., histamine and S-(5′-adenosyl)-L-homocysteine), two amino acids (i.e., 4-guanidinobutyric acid and glutathione), one carbohydrate (i.e., methyl-*D*-galactopyranoside), and two fatty acids (i.e., *D*-erythro-dihydrosphingosine and sebacic aid), displayed higher contents in L_FL. The peak area of the amino acid derivative 4-guanidinobutyric acid in L_FL (375.73 × 10^4^) was 6.6 times higher than that of H_FL (56.87 × 10^4^), the peak area of the alkaloid *S*-(5′-adenosyl)-*L*-homocysteine in L_FL (244.13 × 10^4^) was 2.68 times larger than that of H_FL (90.81 × 10^4^), and methyl-*D*-galactopyranoside in L_FL (12.83 × 10^4^) was 3.65 times greater than that of H_FL (3.51 × 10^4^). There were eight saccharides (i.e., adonitol and *D*-(+)-xylose); six fatty acids (e.g., 2-phospho-*D*-glycerate, 3-phospho-*D*-glycerate, and 3-phosphoglyceric acid); three phenolic acids (4-hydroxyphenylacetic acid, homogentisic acid, and p-coumaric acid); eight TCA-related products (i.e., *D*-glucose 6-phosphate, *D*-fructose 6-phosphate, and *D*-galactose 1-phosphate); and three alkaloids (i.e., cytidine and guanosine 5′-monophosphate) had higher relative contents in H_FL. Among them, the peak area of 5′-deoxy-5′-(methylthio) adenosine in H_FL (474.44 × 10^4^) was three times more abundant than that of L_FL (157.70 × 10^4^), and the peak area of phenolic acid p-coumaric acid reached 682.86 × 10^4^, which was 3.25 times higher than that of L_FL (209.52 × 10^4^). Furthermore, the results showed that saccharides and fatty acids were more abundant in H_FL, while higher alkaloids were confirmed in L_FL.

These findings indicate that tea plants from different growing locations exhibit distinct metabolic characteristics. High-altitude conditions favored the accumulation of saccharides, fatty acids, and aroma precursors, likely due to enhanced photosynthetic activity and stress adaptation [32]. In contrast, alkaloids and amino acid derivatives were more concentrated in L_FL, potentially reflecting increased metabolic flux through nitrogen pathways under warmer, lower-elevation conditions. This divergence aligns with previous studies showing that higher saccharide levels enhance sweetness and aftertaste, while alkaloid enrichment can introduce bitterness or pungency [33]. The overall differences in environmental factors—such as soil fertility, moisture, temperature variation, and light intensity between the two sites—collectively shape their distinct secondary metabolite profiles [34,35].

### 3.5. Dynamics of Flavor-Related Compounds During Green Tea Processing

#### 3.5.1. Flavonoids and Carotenoids Changes During GT Processing

**Figure 2:**
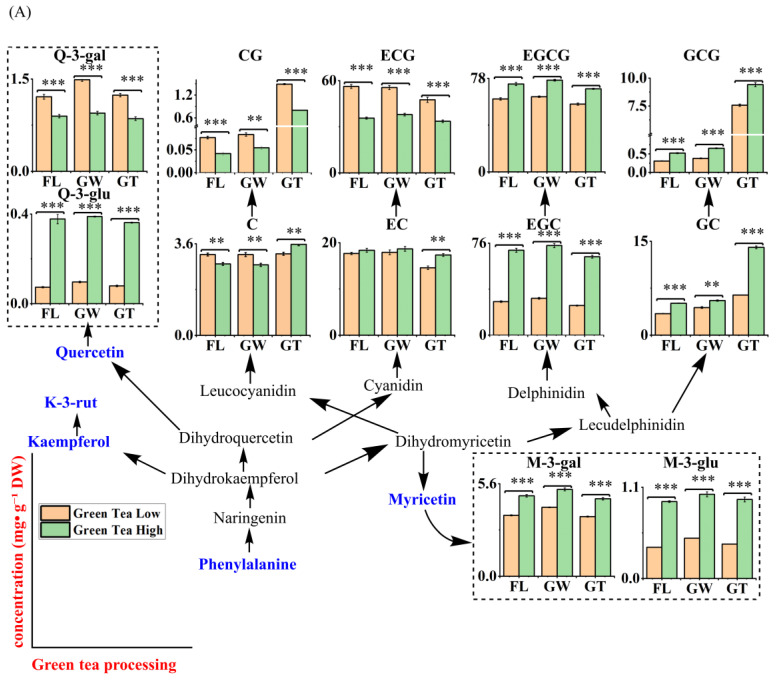

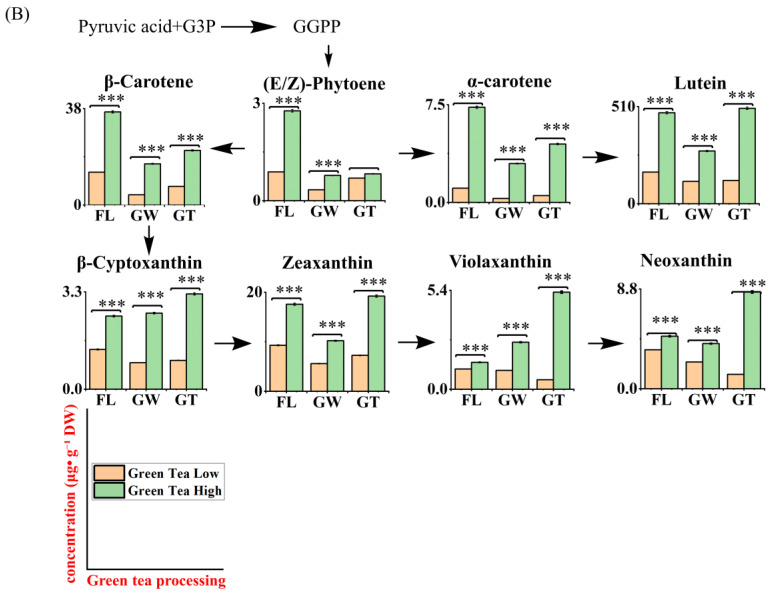

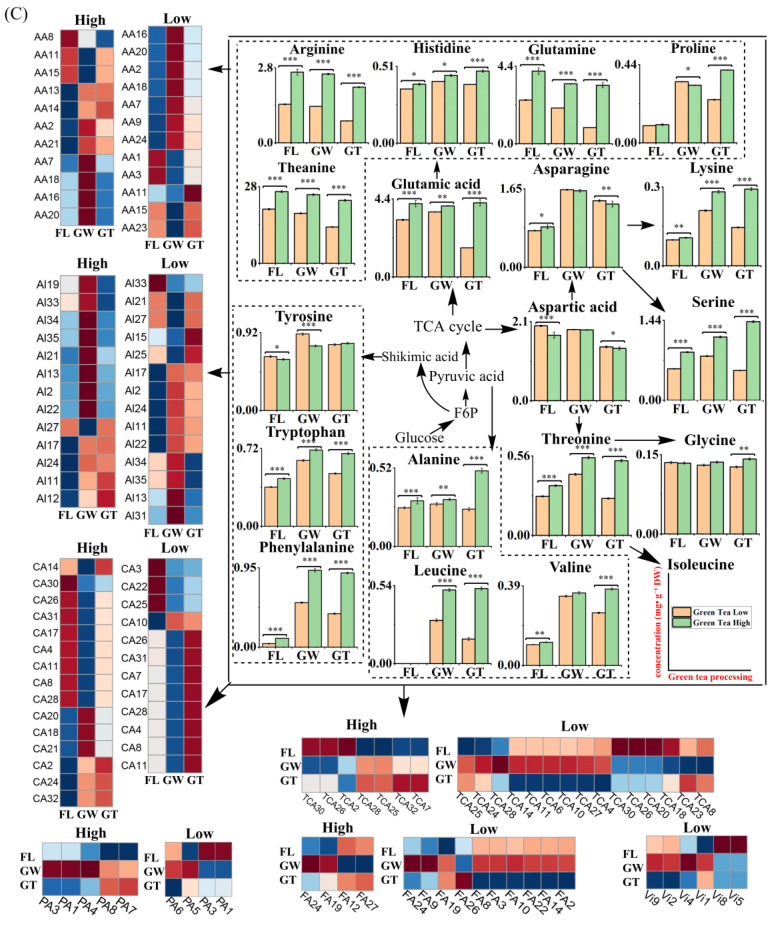
Major flavor compound dynamics illustrated in related pathways during green tea processing using two different altitudes of harvested ‘Huangjinya’ fresh leaves. (**A**) Crucial flavonoid (catechins) changes during green tea processing; (**B**) carotenoid changes during green tea processing; (**C**) amino acid and crucial PM changes during green tea processing. Note: FL, fresh leaves samples; GW, fresh leaves spread for 2 h and collected samples; GT, spread samples processed by fixing, rolling, and drying to make green tea products. Note: *, **, and ***, represents the significant difference that * means <0.05, ** means <0.01, and *** means <0.005.

Targeted analysis of major flavonoids and carotenoids during green tea processing were applied, and the results are sorted into Tables S2 and S3. To achieve a direct view of their changes, an integrative pathway of the pigments in tea plants is presented in Figure 2. It was absolute that, during green tea processing of high-altitude leaves, major catechins such as EGCG and EGC showed a slight increase during the withering step (H_GW) before decreasing in the final product (H_GT) relative to the fresh leaves (H_FL). In contrast, minor catechins like GC and GCG increased substantially in the final tea (Table S2; Figure 2A). In detail, EGCG (73.39 to 69.35 mg·g^−1^ DW and 60.92 to 56.55 mg·g^−1^ DW in H_GT and L_GT, respectively), EGC (70.20 to 64.87 mg·g^−1^ DW and 27.72 to 24.48 mg·g^−1^ DW in H_GT and L_GT, respectively), and ECG (35.68 to 33.64 mg·g^−1^ DW and 56.25 to 47.60 mg·g^−1^ DW in H_GT and L_GT, respectively) remained at similar stable levels in GT products, with a slight increase during spreading, as shown in Figure 2A. It was interesting that GC (5.11 to 14.03 mg·g^−1^ DW and 3.44 to 6.42 mg·g^−1^ DW in H_GT and L_GT, respectively) and GCG (0.52 to 9.42 mg·g^−1^ DW and 0.31 to 7.57 mg·g^−1^ DW in H_GT and L_GT, respectively) increased sharply after the drying step. Considering the concentrations of M-3’-glu, Q-3-gal, K-3-rut, and Q-3-glu (<1.5 mg·g^−1^ DW), their dynamics during processing were mild.

In contrast, carotenoids were highly responsive to processing (Figure 2B). All major carotenoid individuals, especially β-carotene and lutein, showed a similar pattern during low-altitude leaves processing, which gradually declined after the spreading and drying stage from 12.97 to 7.38 μg·g^−1^ DW and 166.35 to 122.37 μg·g^−1^ DW. Carotenoid changes during processing also differed between high- and low-altitude site fresh leaves (Figure 2B). In high-altitude site tea leaves, seven carotenoids decreased significantly after processing. β-carotene (from 36.72 μg·g^−1^ to 21.55 μg·g^−1^) and α-carotene (from 7.31 μg·g^−1^ to 4.49 μg·g^−1^) showed the most pronounced reductions, decreasing nearly by half. In contrast, eleven other carotenoids, such as lutein palmitate (from 0.15 μg·g^−1^ to 12.15 μg·g^−1^), zeaxanthin (from 17.58 μg·g^−1^ to 19.22 μg·g^−1^), and lutein (from 477.74 μg·g^−1^ to 501.25 μg·g^−1^), increased significantly after processing. These trends suggest that the drying process may enhance the stability or extractability of certain carotenoids while promoting the degradation of others. In contrast, carotenoids from low-altitude site leaves showed the opposite trend: most gradually declined during processing and reached their lowest levels in the final tea product.

#### 3.5.2. Dynamics of AAs and PMs

AAs and VIP > 1 PMs were selected and their dynamics during GT crucial processing steps were monitored (Tables S1 and S4). An integrative figure was illustrated, as shown in Figure 2C. Theanine (19.62 to 13.15 mg·g^−1^ DW), glutamine (2.47 to 0.91 mg·g^−1^ DW), glutamic acid (3.21 to 1.62 mg·g^−1^ DW), aspartic acid (1.99 to 1.43 mg·g^−1^ DW), and arginine (1.44 to 0.82 mg·g^−1^ DW) were the five major Aas and exhibited apparent decreases in L_GT. Conversely, the remaining 11 amino acids showed slight accumulation in GT, such as asparagine (0.77 to 1.40 mg·g^−1^ DW), which accumulated in green tea and consistently exhibited higher levels than in the green tea processing group. Considering the higher contents of AAs in the H_FLs, their changes were milder during the processing steps compared to the L_FLs processing, especially theanine (25.69 to 22.78 mg·g^−1^ DW).

During low-altitude GT processing, PMs significantly changed, including 12 AA derivates, 14 alkaloids, 12 carbohydrates, 4 phenolic acids, 15 TCA-related products, 10 fatty acids, and 6 vitamins. AA2, 7, 9, 11, 15, 16, 20, 23, and 24 apparently exhibited increases in L_GT, especially AA11 with a 1.93-fold increase compared to the L_FLs. As for the carbohydrates, most of them had a >1.1-fold increase in L_GW (CA4, 7, 8, 11, 17, 26, 28, and 31) compared to L_FL. Furthermore, concerning the TCA-related intermediates, tricarboxylic acid correlated with shikimic acid in L_GW (62.79 × 10^4^) was 1.1 times higher than that in L_FL (58.86 × 10^4^). As for the alkaloids, most of them achieved an increase with an average 1.63-fold change in L_GW (Al2, 11, 13, 22, 24, 31, 34, and 35), and most of the fatty acids achieved a 1.16-fold change increase in L_GW (FA2, 3, 8, 9, 10, 14, 22, and 24). In addition, the phenolic acids and vitamins changed significantly.

During high altitude GT processing, PMs significantly changes, including 11 AAs derivates, 13 alkaloids, 15 carbohydrates, 5 phenolic acids, 7 TCA-related products, and 4 fatty acids. AA7, 16, 18, and 20 apparently exhibited an increase in H_GW of 1.31-fold compared to H_FLs. As for the carbohydrates, CA18, 20, and 21 exhibited a 1.3-fold increase in H_GW; CA2, 14, 24, and 32 achieved a 1.27-fold increase in H_GT compared to H_FLs. Furthermore, TCA-related intermediates, for instance, TCA7, 25, 28, and 32, exhibited an increase in H_GT (1.29-fold increase) compared to H_FLs. As for alkaloids, most of them achieved a >2-fold increase in L_GW (Al2, 13, 19, 21, 22, 33, 34, and 35), especially Al21 with a 10-fold increase, compared to H_FLs. Fatty acids FA 12, 19, and 24 achieved an increase in H_GT of 1.87-fold.

It was reported that free AAs lay the foundation of umami and sweet tastes in tea soup [36], and higher AAs remaining in H_GT could offer a clear explanation for its premium quality. However, our results showed a different conclusion, with our previous study, that the breakdown of proteins by peptidase could, to a great degree, promote free AAs [37]. Due to the optimized cultivation environment with high altitude [38], it was more advantageous for free AA biosynthesis and accumulation, successively maintaining higher levels in H_GT compared to L_FLs, according to our results. In addition, previous studies have demonstrated that theanine is directly biosynthesized from glutamic acid and ethylamine by theanine synthetase in roots, successively transported to tender leaves waiting for processing [39]. Furthermore, phenylalanines function as crucial precursors to produce flavonoids, which undergo a series of enzymatic reactions [40].

### 3.6. Dynamics of Flavor-Related Compounds During Black Tea Processing

#### 3.6.1. Flavonoids and Carotenoids Changes

**Figure 3:**
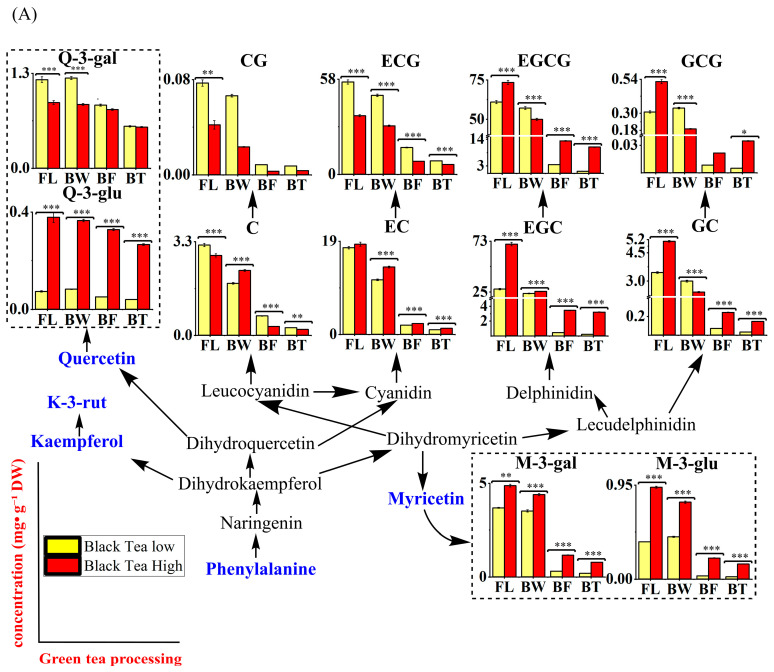

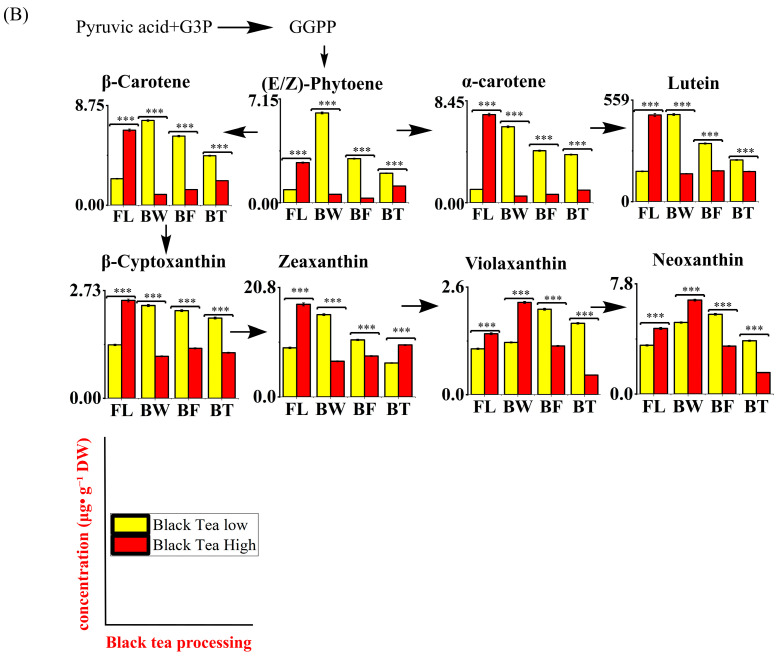

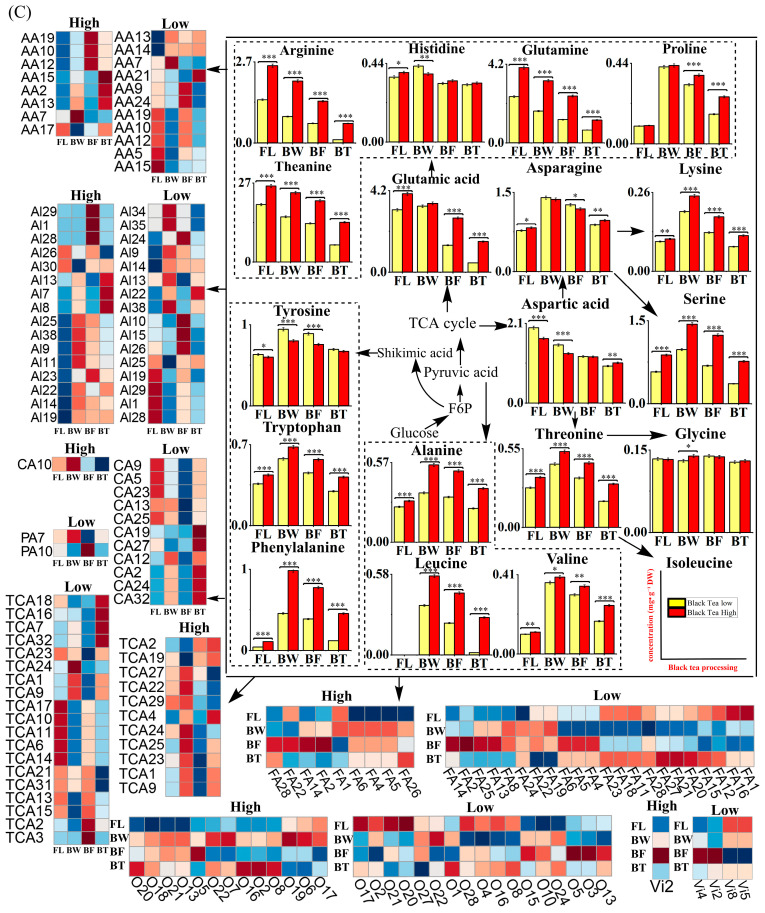
Major flavor compound dynamics illustrated in related pathways during black tea processing using two different altitudes of harvested ‘Huangjinya’ fresh leaves. (**A**) Crucial flavonoid (catechins) changes during green tea processing; (**B**) carotenoid changes during green tea processing; (**C**) amino acid and crucial PM changes during green tea processing. Note: FL, fresh leaves samples; BW, fresh leaves withered for 12 h and collected samples; BF, withered samples processed by rolling and fermentation; BT, fermented samples dried to make black tea products. Note: *, **, and ***, represents the significant difference that * means <0.05, ** means <0.01, and, *** means <0.005.

Targeted analysis of the major flavonoids and carotenoids during black tea processing was applied, and the results are sorted into Tables S5 and S6. To achieve a direct view of their changes, an integrative pathway of pigments in tea plants is presented in Figure 3, showing the accumulation of catechins in black tea fresh leaves and sudden decline during processing. Most of catechins largely accumulate in black tea fresh leaves and experience a sudden decline during processing, especially major catechins EGCG (73.39 to 10.76 mg·g^−1^ and 60.92 to 0.85 mg·g^−1^ in H_BT and L_BT, respectively), EGC (70.20 to 3.22 and 27.72 to 0.22 mg·g^−1^ in H_BT and L_BT, respectively), and ECG (35.68 to 6.03 and 56.25 to 8.22 mg·g^−1^ in H_BT and L_BT, respectively), which remained at similar stable levels in BT products and decreased sharply during the fermentation, as shown in Figure 3A. EC (18.38 to 1.23 and 17.68 to 0.95 mg·g^−1^ in H_BT and L_BT, respectively) and GC (5.11 to 0.15 and 3.44 to 0.03 mg·g^−1^ in H_BT and L_BT, respectively) were interesting. Considering the concentrations of M-3′-glu, Q-3-gal, K-3-rut, and Q-3-glu (<1.5 mg·g^−1^), their dynamics during processing were mild.

Compared to green tea, carotenoids in black tea also exhibit complex accumulation patterns influenced by altitude, yet these patterns are diametrically opposed (as shown in Figure 3B). All major carotenoid individuals, especially β-carotene and lutein, showed a similar pattern during low-altitude leaves processing, which gradually increased after the spreading and drying stage from 12.97 to 24.22 and 166.35 to 229.81 μg·g^−1^. However, the results showed a different tendency from carotenoids originating from high-altitude site FLs during processing (Figure 3B). Carotenes, especially β-carotene (36.72 to 11.99 μg·g^−1^) and α-carotene (7.31 to 1.03 μg·g^−1^), decreased by nearly 77% in H_BT when compared to H_FLs. It was a novel finding that lutein dimyristate and zeaxanthin showed decreases in H_BT. These trends suggest that high-altitude site BT undergoes a greater oxidative degradation of pigments during prolonged fermentation.

#### 3.6.2. Dynamics of AAs and PMs During BT Processing

AAs and VIP > 1 PMs were selected, and their dynamics during BT crucial processing steps were monitored (Tables S1 and S7). An integrative figure was illustrated, as shown in Figure 3C. Theanine (25.96 to 13.58 mg·g^−1^), glutamine (4.02 to 1.21 mg·g^−1^), glutamic acid (4.03 to 1.57 mg·g^−1^), aspartic acid (1.70 to 1.06 mg·g^−1^), and arginine (2.57 to 0.65 mg·g^−1^) were the five major Aas and exhibited apparent decreases in L_BT. Opposite that, phenylalanine (0.11 to 0.45 mg·g^−1^) accumulated in BW and maintained higher levels the H_BT. The contents of AAs in L_FLs were lower compared to H_FLs, and their patterns of change during the processing steps were similar; however, they decreased more drastically, especially theanine (19.62 to 5.82 mg·g^−1^).

During low-altitude BT processing, PMs significantly change, including 11 AA derivates, 16 alkaloids, 11 carbohydrate, 2 phenolic acids, 19 TCA-related products, 22 fatty acids, and 4 vitamins. AA13, AA14, and AA21 apparently exhibited an increase in L_BT, especially AA14 with a 4.9-fold increase compared to L_FLs. As for carbohydrates, most of them achieved an increase in L_BT, shown in CA 2, 12, 19, 24, 27, and 32 with a 1.95-fold change. Furthermore, TCA-related intermediates TCA 7, 16, 18, and 32 in L_BT were 1.78 times higher than those in L_FL. As for alkaloids, some of them achieved an increase in L_BW (Al 9, 13, 14, 22, 34, 35, and 38), with a 1.93-fold change. Fatty acids, for instance, FA 2, 4, 5, 6, 8, 13, 14, 24, and 25, achieved 1.66-fold change. In addition, phenolic acids and vitamins changed significantly.

It was similar during high-altitude site BT processing. PMs significantly changed, including 8 AA derivates, 16 alkaloids, 1 carbohydrate, 11 TCA-related products, 9 fatty acids, and 1 vitamin. AA 2, 13, 15, and 19 exhibited clear increases in H_BT, with a 1.23-fold increase compared to H_FLs. As for carbohydrates, CA 10 achieved an increase in H_BW, with a 1.66-fold increase compared to H_FLs. Furthermore, TCA 1, 9, 22, 23, 24, 25, 27, and 29 also exhibited increased tendencies in H_BW, with a 1.33-fold increase compared to H_FLs. Al 9, 11, 14, 19, 22, 25, and 38 achieved a 4.17-fold change compared to H_FLs, as in L_BW. Fatty acids in H_BW, like FA 1, 4, 5, 6, 10, 22, 14, and 2, exhibited a 4.76-fold change. In addition, phenolic acids and vitamins changed significantly, especially vitamins (α-tocopherol), which increased 2.3-fold in H_BF.

These changes indicate intense metabolic turnover during BT processing, particularly under oxidative conditions. High-altitude site BT showed better retention of amino acids but greater pigment degradation, while low-altitude site BT accumulated more secondary metabolites like alkaloids and fatty acids, which may contribute to aroma complexity and mouthfeel.

### 3.7. Correlation Between Pigments and Major Taste Compounds and Their Contribution to Quality

**Figure 4:**
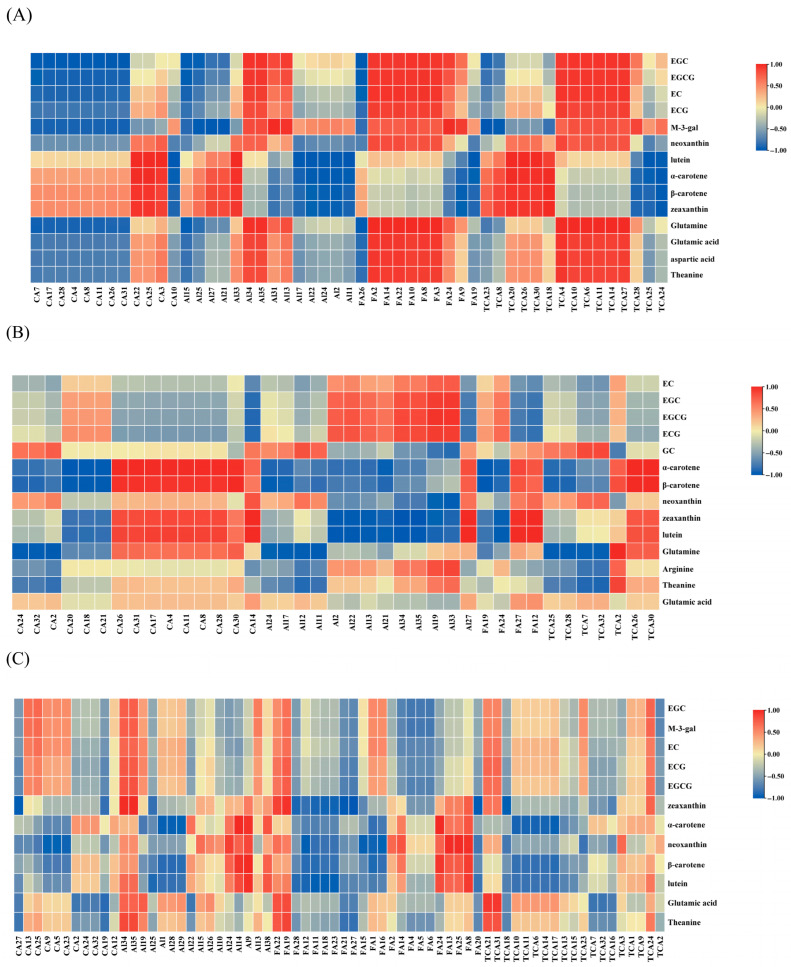

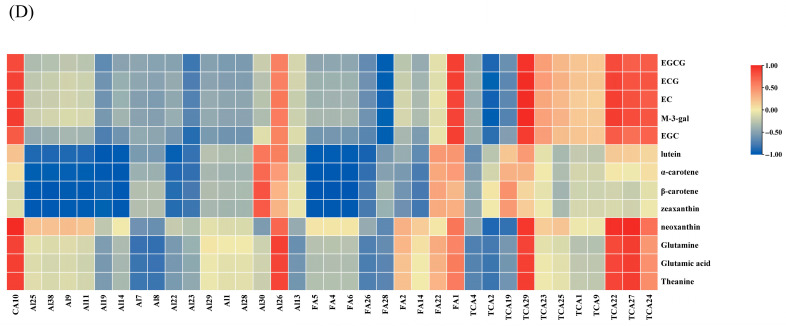
Correlation analyses between crucial pigments and primary metabolites. (**A**) Crucial compounds correlation analysis of low-altitude site green tea; (**B**) crucial compounds correlation analysis of high-altitude site green tea; (**C**) crucial compounds correlation analysis of low-altitude site black tea; (**D**) crucial compounds correlation analysis of high-altitude site black tea.

To better understand the underlying metabolic mechanisms contributing to tea quality formation, Pearson correlation analyses were performed between pigments (flavonoids and carotenoids) and major flavor compounds—including amino acids (AAs) and primary metabolites (PMs)—in green and black teas made from high- and low-altitude site ‘Huangjinya’ leaves. All metabolites included met the criteria of VIP > 1 and *p* < 0.01 (Figure 4). While these correlation analyses provide valuable insights into the potential metabolic relationships shaping tea flavor, the results should be interpreted with caution due to the limited number of biological replicates (*n* = 3). These findings highlight strong associations that warrant confirmation in future studies with larger sample sizes.

#### 3.7.1. Metabolite Correlation Patterns and Flavor Implications in Green Tea Prepared from ‘Huangjinya’

In low-altitude site green tea (L_GT), 51 differential PMs were used in the analysis, including 12 carbohydrates (e.g., CA 3, 4, 8, 26, and 31); 14 alkaloids (e.g., Al 34, 35, 31, 13); 10 fatty acids (e.g., FA 9 and FA 19); and 15 TCA-related intermediates (e.g., TCA 10, 26, and 30) (Figure 4A). The five major flavonoids—EGC, EGCG, EC, ECG, and M-3-gal—showed strong positive correlations with alkaloids, particularly the synergy between M-3-gal and Al 31 (r = 0.99). They were also positively correlated with most fatty acids, except FA 26 (r = −0.91), and with tricarboxylic acid intermediates, especially EGC–TCA 10 (r = 0.96). In contrast, flavonoids were negatively correlated with most carbohydrates, with EGC and CA 4, 8, 11, 26, and 31 displaying strong opposition (r = −0.97). Carotenoids such as neoxanthin, lutein, α-carotene, β-carotene, and zeaxanthin were positively correlated with most carbohydrates, particularly CA 22, CA 25, and CA 3 (r = 0.90), but showed negative correlations with most alkaloids (Al 17, 24, and 22) and fatty acids (FA 9 and FA 19). A novel correlation was observed between lutein and TCA intermediates (TCA 20, 26, 30, and 18), while α-carotene was negatively correlated with TCA 24 and 27 (r = −0.97) but positively correlated with most other TCAs. For amino acids, glutamic acid and theanine (content > 2 mg/g) exhibited negative correlations with most carbohydrates and alkaloids and positive correlations with most fatty acids and TCA intermediates. Glutamic acid showed a strong correlation with TCA 4 (r = 0.95).

In high-altitude site green tea (H_GT), 39 metabolites were selected, including 15 carbohydrates (e.g., CA 2, 4, 11, and 30); 13 alkaloids, 4 fatty acids, and 7 TCA intermediates (Figure 4B). Flavonoids (EGCG, EGC, EC, ECG, and GC) displayed positive correlations with most alkaloids, especially EGCG–Al 33 (r = 0.95), and negative correlations with most carbohydrates, except for GC–CA 2 (r = 0.76). They were positively associated with most fatty acids but negatively correlated with TCA-related compounds. Carotenoids showed a different correlation pattern. β-carotene was positively correlated with CA 26, 30, and 31 (r = 0.97) but negatively correlated with CA 18, 20, and 21 (r = −0.97). Zeaxanthin and lutein were strongly positively correlated with Al 27 (r = 0.99). A newly observed pattern showed that α- and β-carotene were positively correlated with TCA 26 and 30 (r = 0.99), while β-carotene and TCA 25 and 28 were negatively correlated (r = −0.90).

These correlations reflect the biochemical basis of the mild, fresh, and umami-rich flavor of green tea, particularly from high-altitudes site. The strong positive relationship between flavonoids and alkaloids, fatty acids, and TCA intermediates suggests a coordinated metabolic environment that favors stable antioxidant profiles and energy balance, contributing to smoother astringency and depth of flavor. Meanwhile, amino acids like theanine and glutamic acid, correlated positively with TCAs and lipids but negatively with sugars, may reinforce umami taste and sweetness, rather than bitterness. The negative correlation between flavonoids and sugars could also indicate a shift away from basic carbon storage toward secondary metabolite synthesis in quality-driven green teas. These findings support the sensory perception that high-altitude site green teas possess higher taste complexity and delicacy.

#### 3.7.2. Metabolite Correlation Patterns and Flavor Implications in Black Tea Prepared from ‘Huangjinya’

In low-altitude site black tea (L_BT), 68 metabolites were included in the correlation analysis, including 11 carbohydrates, 16 alkaloids, 22 fatty acids, and 19 TCA-related intermediates (Figure 4C). Five key flavonoids (EGC, EGCG, EC, ECG, and M-3-gal) demonstrated strong positive correlations with alkaloids, especially EGCG–Al 34/35 (r = 0.80). They were also positively associated with several TCA intermediates and carbohydrates, such as M-3-gal–TCA 24 (r = 0.71), but negatively correlated with fatty acids, with notable opposition between M-3-gal/EGC and FA 4 (r = −0.79). Carotenoids were negatively correlated with most carbohydrates and fatty acids, for example, zeaxanthin–CA 27 (r = −0.96) and zeaxanthin–FA 27 (r = −0.99). In contrast, they were positively associated with alkaloids, particularly zeaxanthin–Al 34 (r = 0.98). α-carotene showed a strong negative correlation with TCA 17 (r = −0.94).

In high-altitude site black tea (H_BT), 37 metabolites were analyzed, including 1 carbohydrate (CA 10), 16 alkaloids, 9 fatty acids, and 11 TCA-related intermediates (Figure 4D). Flavonoids were negatively correlated with most alkaloids, such as EGC–Al 23 (r = −0.86), but positively correlated with CA 10 (r = 0.86) and TCA 29 (r = 0.97). They were also negatively associated with fatty acid FA 28 (r = −0.94). Carotenoids, including neoxanthin, lutein, α-carotene, β-carotene, and zeaxanthin, were generally negatively associated with alkaloids and fatty acids, particularly α-carotene–FA 4/5/6 (r = −0.99) and β-carotene–Al 38 (r = −0.99). Neoxanthin showed negative correlation with TCA 2 (r = −0.90) and positive correlation with CA 10 (r = 0.99). Major amino acids (theanine, glutamic acid, and glutamine) in H_BT were positively associated with CA 10 (r = 0.89) but negatively correlated with alkaloids, fatty acids, and TCA compounds, particularly theanine–TCA 29 (r = −0.91).

The correlation patterns observed in the black tea samples suggest distinct metabolic strategies that shape their stronger taste and more complex aroma profiles. In low-altitude site BT, the positive relationship between flavonoids, alkaloids, and TCAs points to a fermentation-driven transformation of catechins into theaflavins and thearubigins, which underpin the brisk and sweet flavor of black tea. Meanwhile, negative correlations with fatty acids may suggest these compounds are oxidized or transformed into aroma-active volatiles. In contrast, high-altitude site BT retained more amino acids and sugars (CA 10), potentially contributing to milder sweetness and reduced bitterness, despite severe carotenoid and flavonoid degradation. The negative correlation between pigments and alkaloids/fatty acids in H_BT further indicates that oxidative pathways were more active, possibly explaining the lighter taste and aroma typical of high-altitude site black teas. It is important to note that, while altitude is a major distinguishing feature of the two cultivation sites, other covariates such as soil composition and specific microclimates could also contribute to the observed metabolic profiles. Future studies are planned to disentangle these variables.

## 4. Conclusions

This study investigated how cultivation site and processing influence the accumulation and transformation of pigments and flavor-related metabolites in the yellow-leaf tea cultivar ‘Huangjinya’. High-altitude site fresh leaves exhibited significantly higher levels of carotenoids and flavonoids, as well as greater retention of theanine and glutamic acid during processing. These compounds were strongly associated with the umami and mellow flavor characteristic of high-altitude site green tea. In contrast, low-altitude site samples, particularly black teas, showed increased levels of alkaloids and fatty acids, contributing to a more robust taste and complex aroma. Catechins remained relatively stable during green tea processing but were largely degraded in black tea. Carotenoid degradation patterns varied by cultivation site, with higher losses observed in high-altitude site cultivation site black tea. Correlation analysis revealed that pigments were closely linked with amino acids, fatty acids, and TCA intermediates, suggesting coordinated metabolic responses. These findings provide a biochemical foundation for improving tea quality through site-specific cultivation and processing strategies.

Based on the aforementioned research findings, this study also has certain limitations. The current research design only includes observational data from two representative altitudinal origins and a single production season. While it effectively reveals the dynamic changes in metabolites associated with origin characteristics and processing, it remains insufficient for comprehensively elucidating the complex mechanisms of interactions among multiple environmental factors. Therefore, in the Discussion and Outlook section, we propose a follow-up research plan: Through systematic replication across multiple origins and seasons, combined with continuous environmental factor monitoring and orthogonal experimental methods such as transcriptomics, we will delve deeper into elucidating the regulatory mechanisms governing metabolite accumulation and transformation. This will provide a more comprehensive scientific basis for systematically deciphering the formation of tea quality.

## Figures and Tables

**Table 1:** Constituents and concentrations of flavonoids and carotenoids in ‘Huangjinya’ fresh leaves cultivated at two altitudes sites.

Compounds	Fresh Tea Leaves		*p* Value
	Low	High	
Flavonoids	mg·g^−1^ DW		
EGC	27.72 ± 0.67	70.20 ± 1.65	<0.001
EGCG	60.92 ± 0.99	73.39 ± 1.29	<0.001
ECG	56.25 ± 1.28	35.68 ± 0.64	<0.001
EC	17.68 ± 0.25	18.38 ± 0.42	NS
GC	3.44 ± 0.04	5.11 ± 0.05	<0.001
C	3.18 ± 0.06	2.81 ± 0.06	<0.01
GCG	0.31 ± 0.01	0.52 ± 0.02	<0.001
CG	0.08 ± 0.00	0.04 ± 0.00	<0.001
Myricetin-3-galactoside (M-3-gal)	3.69 ± 0.03	4.87 ± 0.08	<0.001
Myricetin-3-glucoside (M-3-glu)	0.38 ± 0.00	0.93 ± 0.01	<0.001
Myricetin (M)	0.08 ± 0.00	0.12 ± 0.01	<0.01
Kaempferol-3-rutinoside (K-3-rut)	0.23 ± 0.01	0.58 ± 0.02	<0.001
Kaempferol (K)	0.01 ± 0.00	0.00 ± 0.00	<0.001
Quercetin-3-galactoside (Q-3-gal)	1.21 ± 0.04	0.90 ± 0.03	<0.001
Quercetin-3-glucoside (Q-3-glu)	0.07 ± 0.00	0.38 ± 0.02	<0.001
Quercetin (Q)	0.00 ± 0.00	0.00 ± 0.00	<0.001
Flavonoids in total	175.25 ± 3.20	213.91 ± 3.78	<0.001
Carotenoids	μg·g^−1^ DW		
α-Carotene	1.10 ± 0.01	7.31 ± 0.09	<0.001
β-Carotene	12.97 ± 0.17	36.72 ± 0.47	<0.001
(E/Z)-Phytoene	0.89 ± 0.01	2.77 ± 0.04	<0.001
Lutein palmitate	0.12 ± 0.00	0.15 ± 0.00	<0.001
Zeaxanthin	9.31 ± 0.12	17.58 ± 0.23	<0.001
Violaxanthin	1.11 ± 0.01	1.47 ± 0.02	<0.001
Neoxanthin	3.45 ± 0.04	4.64 ± 0.06	<0.001
Lutein	166.35 ± 2.14	477.74 ± 6.14	<0.001
β-Cryptoxanthin	1.35 ± 0.02	2.48 ± 0.03	<0.001
8′-Apo-beta-carotenal	0.02 ± 0.00	0.03 ± 0.00	<0.01
Canthaxanthin	0.00 ± 0.00	0.00 ± 0.00	<0.001
Echinenone	0.01 ± 0.00	0.01 ± 0.00	<0.001
β-Citraurin	0.00 ± 0.00	0.01 ± 0.00	<0.001
Lutein myristate	0.11 ± 0.00	0.25 ± 0.00	<0.001
Violaxanthin dibutyrate	0.03 ± 0.00	0.01 ± 0.00	<0.001
α-Cryptoxanthin	0.13 ± 0.00	0.53 ± 0.01	<0.001
Lutein dilaurate	0.03 ± 0.00	0.06 ± 0.00	<0.001
Lutein dimyristate	0.05 ± 0.00	0.10 ± 0.00	<0.001
Carotenoids in total	197.02 ± 2.53	551.88 ± 7.09	<0.001
Total pigments	327.27 ± 2.83	765.79 ± 6.67	<0.001

Note: Mean values listed in the table were all from three replicates. Statistical comparisons were based on differences between low and high altitudes. Low altitude = ~80 m and high altitude = ~600 m.

**Table 2:** Constituents and concentrations of amino acids in ‘Huangjinya’ fresh leaves cultivated at two altitudes sites (mg·g^−1^ DW).

Amino Acids	Fresh Tea Leaves		*p* Value
	Low	High	
Phenylalanine	0.04 ± 0.00	0.11 ± 0.00	<0.001
Leucine	nd	nd	ns
Tryptophan	0.36 ± 0.01	0.44 ± 0.01	<0.001
Valine	0.10 ± 0.00	0.11 ± 0.00	<0.01
Proline	0.10 ± 0.00	0.10 ± 0.00	0.090
Tyrosine	0.63 ± 0.01	0.60 ± 0.01	<0.05
Alanine	0.25 ± 0.00	0.29 ± 0.00	<0.001
Threonine	0.28 ± 0.00	0.35 ± 0.01	<0.001
Glycine	0.13 ± 0.00	0.13 ± 0.00	ns
Glutamine	2.47 ± 0.04	4.02 ± 0.07	<0.001
Serine	0.57 ± 0.01	0.88 ± 0.01	<0.001
Glutamic acid	3.21 ± 0.05	4.03 ± 0.07	<0.001
Asparagine	0.77 ± 0.01	0.83 ± 0.01	<0.05
Aspartic acid	1.99 ± 0.03	1.70 ± 0.03	<0.001
Histidine	0.36 ± 0.01	0.39 ± 0.01	<0.001
Arginine	1.44 ± 0.02	2.57 ± 0.04	<0.001
Lysine	0.10 ± 0.00	0.11 ± 0.00	<0.01
Isoleucine	0.01 ± 0.00	0.01 ± 0.00	<0.01
Theanine	19.62 ± 0.33	25.96 ± 0.44	<0.001
in total	32.44 ± 0.55	42.63 ± 0.72	<0.001

Note: Mean values listed in the table were all from three replicates. Statistical comparisons were based on differences between low and high altitudes. Low altitude = ~80 m and high altitude = ~600 m. nd represented the value of the amino acid being lower than the analyzed limitation.

## Data Availability

The original contributions presented in this study are included in the article/Supplementary Materials. Further inquiries can be directed to the corresponding author.

## Supplementary Materials

The following supporting information can be downloaded at https://www.mdpi.com/article/10.3390/foods14203575/s1: Table S1. Primary identification of ‘Huangjinya’ fresh leaves from two altitudes and their changes during green tea and black tea production; Table S2. Flavonoid changes during green tea processing using different altitude cultivated fresh leaves (mg·g^−1^ DW); Table S3. Carotenoid changes during green tea processing using different altitude cultivated fresh leaves (μg·g^−1^ DW); Table S4. Amino acid changes during green tea processing using different altitude cultivated fresh leaves (mg·g^−1^ DW); Table S5. Flavonoid changes during black tea processing using different altitude cultivated fresh leaves (mg·g^−1^ DW); Table S6. Carotenoid changes during black tea processing using different altitude cultivated fresh leaves (μg·g^−1^ DW); Table S7. Amino acid changes during black tea processing using different altitude cultivated fresh leaves (mg·g^−1^ DW).
