# Supplementary material for: Comparative Analysis of Flavonoids, Carotenoids, and Major Primary Compounds in Site-Specific Yellow-Leaf Tea and Their Dynamic Alterations During Processing
Source: Foods. 2025 Oct 21;14(20):3575. doi: 10.3390/foods14203575 (PMC12563805; doi:10.3390/foods14203575)
Supplement: Supplementary file 1 [file foods-14-03575-s001.zip › SI.pdf]

Table S2 Flavonoids changes during green tea processing using different altitude cultivated fresh leaves (mg•g^{-1} DW)

Flavonoids	Green tea prepared from high-altitude cultivated fresh leaves			Green tea prepared from low-altitude cultivated fresh leaves		
	FL	GW	GT	FL	GW	GT
EGC	70.20±1.65	74.08±1.75	64.87±1.17	27.72±0.67	30.48±0.72	24.48±0.44
EGCG	73.39±1.29	76.55±0.73	69.35±0.46	60.92±0.99	62.78±0.61	56.55±0.95
ECG	35.68±0.64	37.99±0.88	33.64±0.83	56.25±1.28	55.56±1.29	47.60±1.73
EC	18.38±0.42	18.67±0.53	17.36±0.30	17.68±0.25	17.93±0.52	14.60±0.39
GC	5.11±0.05	5.53±0.11	14.03±0.22	3.44±0.04	4.42±0.15	6.42±0.01
M-3-gal	4.87±0.08	5.26±0.09	4.69±0.07	3.69±0.03	4.17±0.03	3.61±0.03
C	2.81±0.06	2.77±0.06	3.58±0.05	3.18±0.06	3.18±0.08	3.21±0.06
M-3'-glu	0.93±0.01	1.01±0.03	0.95±0.03	0.38±0.00	0.49±0.00	0.42±0.00
GCG	0.52±0.02	0.66±0.01	9.42±0.18	0.31±0.01	0.38±0.01	7.57±0.11
Q-3-gal	0.90±0.03	0.95±0.03	0.86±0.03	1.21±0.04	1.49±0.03	1.24±0.03
K-3-rut	0.58±0.02	0.60±0.00	0.6±0.01	0.23±0.01	0.27±0.00	0.25±0.00
Q-3-glu	0.38±0.02	0.39±0.01	0.36±0.00	0.07±0.00	0.10±0.00	0.08±0.00
Myricetin	0.12±0.01	0.13±0.00	0.22±0.00	0.08±0.00	0.08±0.00	0.17±0.00
CG	0.04±0.00	0.05±0.00	0.8±0.01	0.08±0.00	0.08±0.00	1.49±0.02
Quercetin	0.00±0.00	0±0.00	0.02±0.00	0.00±0.00	0.00±0.00	0.02±0.00
Kaempferol	0.00±0.00	0.01±0.00	0.02±0.00	0.01±0.00	0.01±0.00	0.02±0.00

Note: Myricetin_3_galactoside, M-3-gal; Myricetin_3'_glucoside, M-3'-glu; Quercetin_3_galactoside, Q-3-gal; Kaempferol_3_rutinoside, K-3-rut; Quercetin_3_glucoside, Q-3-glu

Table S3 Carotenoids changes during green tea processing using different altitude cultivated fresh leaves (μg•g^{-1} DW)

Carotenoids	Green tea prepared from high-altitude cultivated fresh leaves			Green tea prepared from low-altitude cultivated fresh leaves		
	FL	GW	GT	FL	GW	GT
α-Carotene	7.31±0.09	3.00±0.04	4.49±0.06	1.10±0.01	0.31±0.00	0.53±0.01
β-Carotene	36.72±0.47	16.26±0.21	21.55±0.28	12.97±0.17	4.08±0.05	7.38±0.09
(E/Z)-Phytoene	2.77±0.04	0.78±0.01	0.83±0.01	0.89±0.01	0.34±0.00	0.7±0.01
Lutein palmitate	0.15±0.00	6.89±0.09	12.15±0.16	0.12±0.00	0.08±0.00	0.57±0.01
Zeaxanthin	17.58±0.23	10.22±0.13	19.22±0.25	9.31±0.12	5.61±0.07	7.27±0.09
Violaxanthin	1.47±0.02	2.57±0.03	5.3±0.07	1.11±0.01	1.04±0.01	0.53±0.01
Neoxanthin	4.64±0.06	3.99±0.05	8.54±0.11	3.45±0.04	2.37±0.03	1.28±0.02
Lutein	477.74±6.14	277.12±3.56	501.25±6.44	166.35±2.14	117.22±1.51	122.37±1.57
β-Cryptoxanthin	2.48±0.03	2.58±0.03	3.23±0.04	1.35±0.02	0.90±0.01	0.98±0.01
8'-Apo-beta-carotenal	0.03±0.00	0.03±0.00	0.04±0.00	0.02±0.00	0.03±0.00	0.02±0.00
Canthaxanthin	0.00±0.00	0.00±0.00	0±0.00	0.00±0.00	0.01±0.00	0.00±0.00
Echinenone	0.01±0.00	0.04±0.00	0.06±0.00	0.01±0.00	0.03±0.00	0.02±0.00
β-Citraurin	0.01±0.00	0.01±0.00	0.01±0.00	0.00±0.00	0.00±0.00	0.00±0.00
Lutein myristate	0.25±0.00	0.26±0.00	0.39±0.01	0.11±0.00	0.17±0.00	0.18±0.00
Violaxanthin dibutyrate	0.01±0.00	0.01±0.00	0.02±0.00	0.03±0.00	0.04±0.00	0.04±0.00
α-Cryptoxanthin	0.53±0.01	0.23±0.00	0.48±0.01	0.13±0.00	0.06±0.00	0.10±0.00
Lutein dilaurate	0.06±0.00	0.03±0.00	0.03±0.00	0.03±0.00	0.02±0.00	0.04±0.00
Lutein dimyristate	0.10±0.00	0.01±0.00	0.02±0.00	0.05±0.00	0.04±0.00	0.02±0.00

Table S4 Amino acids changes during green tea processing using different altitude cultivated fresh leaves ($mg•g^{-1}$ DW)

Amino acids	Green tea prepared from high-altitude cultivated fresh leaves			Green tea prepared from low-altitude cultivated fresh leaves		
	FL	GW	GT	FL	GW	GT
Phenylalanine	0.11±0.00	0.92±0.02	0.89±0.01	0.04±0	0.53±0.01	0.40±0.01
Leucine	0.00±0.00	0.51±0.01	0.51±0.01	0.00±0.00	0.3±0.01	0.17±0.00
Tryptophan	0.44±0.01	0.7±0.01	0.67±0.01	0.36±0.01	0.6±0.01	0.48±0.01
Valine	0.11±0.00	0.35±0.01	0.37±0.01	0.10±0.00	0.34±0.01	0.26±0.00
Proline	0.10±0.00	0.32±0.01	0.41±0.01	0.10±0.00	0.34±0.01	0.24±0.00
Tyrosine	0.60±0.01	0.75±0.01	0.79±0.01	0.63±0.01	0.89±0.02	0.77±0.01
Alanine	0.29±0.00	0.31±0.01	0.49±0.01	0.25±0.00	0.28±0.00	0.24±0.00
Threonine	0.35±0.01	0.54±0.01	0.52±0.01	0.28±0.00	0.43±0.01	0.26±0.00
Glycine	0.13±0.00	0.13±0.00	0.14±0.00	0.13±0.00	0.13±0.00	0.13±0.00
Glutamine	4.02±0.07	3.4±0.06	3.24±0.05	2.47±0.04	2.03±0.03	0.91±0.02
Serine	0.88±0.01	1.15±0.02	1.43±0.02	0.57±0.01	0.8±0.01	0.54±0.01
Glutamic acid	4.03±0.07	4.01±0.07	4.08±0.07	3.21±0.05	3.67±0.06	1.62±0.03
Asparagine	0.83±0.01	1.59±0.03	1.30±0.02	0.77±0.01	1.63±0.03	1.40±0.02
aspartic acid	1.70±0.03	1.88±0.03	1.32±0.02	1.99±0.03	1.89±0.03	1.43±0.02
Histidine	0.39±0.01	0.45±0.01	0.48±0.01	0.36±0.01	0.41±0.01	0.39±0.01
Arginine	2.57±0.04	2.55±0.04	2.09±0.04	1.44±0.02	1.36±0.02	0.82±0.01
Lysine	0.11±0.00	0.28±0.00	0.29±0.00	0.10±0.00	0.21±0.00	0.14±0.00
isoleucine	0.01±0.00	0.03±0.00	0.03±0.00	0.01±0.00	0.02±0.00	0.02±0.00
Theanine	25.96±0.44	24.87±0.42	22.78±0.38	19.62±0.33	18.1±0.30	13.15±0.22

Table S5 Flavonoids changes during black tea processing using different altitude cultivated fresh leaves (mg•g^{-1} DW)

Flavonoids	Black tea prepared from high-altitude cultivated fresh leaves				Black tea prepared from low-altitude cultivated fresh leaves			
	FL	BW	BF	BT	FL	BW	BF	BT
EGC	70.20±1.65	25.50±0.36	3.45±0.05	3.22±0.05	27.72±0.67	23.56±0.32	0.44±0.01	0.22±0.00
EGCG	73.39±1.29	49.98±0.71	13.29±0.19	10.76±0.15	60.92±0.99	57.13±0.77	3.57±0.05	0.85±0.01
ECG	35.68±0.64	29.57±0.42	7.95±0.11	6.03±0.09	56.25±1.28	48.11±0.65	16.3±0.22	8.22±0.11
EC	18.38±0.42	13.72±0.19	2.21±0.03	1.23±0.02	17.68±0.25	11.16±0.15	1.88±0.03	0.95±0.01
GC	5.11±0.05	2.41±0.03	0.24±0.00	0.15±0.00	3.44±0.04	3.00±0.04	0.07±0.00	0.03±0.00
M-3-gal	4.87±0.08	4.39±0.06	1.16±0.02	0.79±0.01	3.69±0.03	3.52±0.05	0.31±0.00	0.19±0.00
C	2.81±0.06	2.28±0.03	0.32±0.00	0.21±0.00	3.18±0.06	1.84±0.02	0.69±0.01	0.27±0.00
M-3'-glu	0.93±0.01	0.78±0.01	0.21±0.00	0.15±0.00	0.38±0.00	0.43±0.01	0.03±0.00	0.02±0.00
GCG	0.52±0.02	0.19±0.00	0.02±0.00	0.03±0.00	0.31±0.01	0.34±0.00	0.01±0.00	0.00±0.00
Q-3-gal	0.90±0.03	0.87±0.01	0.80±0.01	0.56±0.01	1.21±0.04	1.24±0.02	0.86±0.01	0.57±0.01
K-3-rut	0.58±0.02	0.61±0.01	0.57±0.01	0.53±0.01	0.23±0.01	0.26±0.00	0.2±0.00	0.16±0.00
Q-3-glu	0.38±0.02	0.36±0.01	0.33±0.00	0.27±0.00	0.07±0.00	0.08±0.00	0.05±0.00	0.04±0.00
Myricetin	0.12±0.01	0.23±0.00	0.07±0.00	0.25±0.00	0.08±0.00	0.05±0.00	0.01±0.00	0.02±0.00
CG	0.04±0.00	0.02±0.00	0.00±0.00	0.00±0.00	0.08±0.00	0.07±0.00	0.01±0.00	0.01±0.00
Quercetin	0.00±0.00	0.02±0.00	0.02±0.00	0.04±0.00	0.00±0.00	0.00±0.00	0.01±0.00	0.01±0.00
Kaempferol	0.00±0.00	0.02±0.00	0.03±0.00	0.05±0.00	0.01±0.00	0.01±0.00	0.02±0.00	0.03±0.00

Note: Myricetin_3_galactoside, M-3-gal; Myricetin_3'_glucoside, M-3'-glu; Quercetin_3_galactoside, Q-3-gal; Kaempferol_3_rutinoside, K-3-rut; Quercetin_3_glucoside, Q-3-glu

Table S6 Carotenoids changes during black tea processing using different altitude cultivated fresh leaves (μg•g^{-1} DW)

Carotenoids	Black tea prepared from high-altitude cultivated fresh leaves				Black tea prepared from low-altitude cultivated fresh leaves			
	FL	BW	BF	BT	FL	BW	BF	BT
α-Carotene	7.31±0.09	0.55±0.00	0.68±0.01	1.03±0.01	1.10±0.01	6.3±0.05	4.31±0.03	3.99±0.03
β-Carotene	36.72±0.47	5.24±0.04	7.58±0.06	11.99±0.10	12.97±0.17	41.42±0.33	33.82±0.27	24.22±0.19
(E/Z)-Phytoene	2.77±0.04	0.58±0.00	0.32±0.00	1.16±0.01	0.89±0.01	6.19±0.05	3.04±0.02	2.03±0.02
Lutein palmitate	0.15±0.00	0.07±0.00	0.38±0.00	0.11±0.00	0.12±0.00	0.19±0.00	2.93±0.02	6.01±0.05
Zeaxanthin	17.58±0.23	6.79±0.05	7.78±0.06	9.87±0.08	9.31±0.12	15.64±0.12	10.85±0.09	6.48±0.05
Violaxanthin	1.47±0.02	2.23±0.02	1.17±0.01	0.48±0.00	1.11±0.01	1.27±0.01	2.07±0.02	1.72±0.01
Neoxanthin	4.64±0.06	6.65±0.05	3.39±0.03	1.52±0.01	3.45±0.04	5.07±0.04	5.65±0.04	3.79±0.03
Lutein	477.74±6.14	153.76±1.22	169.38±1.34	165.37±1.31	166.35±2.14	480.08±3.81	319.61±2.54	229.81±1.82
β-Cryptoxanthin	2.48±0.03	1.06±0.01	1.26±0.01	1.15±0.01	1.35±0.02	2.35±0.02	2.22±0.02	2.04±0.02
8'-Apo-beta-carotenal	0.03±0.00	0.03±0.00	0.02±0.00	0.02±0.00	0.02±0.00	0.05±0.00	0.03±0.00	0.03±0.00
Canthaxanthin	0.00±0.00	0.01±0.00	0.00±0.00	0.00±0.00	0.00±0.00	0.00±0.00	0.00±0.00	0.00±0.00
Echinenone	0.01±0.00	0.03±0.00	0.02±0.00	0.01±0.00	0.01±0.00	0.02±0.00	0.03±0.00	0.03±0.00
β-Citraurin	0.01±0.00	0.01±0.00	0.00±0.00	0.00±0.00	0.00±0.00	0.01±0.00	0.00±0.00	0.00±0.00
Lutein myristate	0.25±0.00	0.07±0.00	0.12±0.00	0.20±0.00	0.11±0.00	0.33±0.00	0.36±0.00	0.30±0.00
Violaxanthin dibutyrate	0.01±0.00	0.05±0.00	0.05±0.00	0.03±0.00	0.03±0.00	0.02±0.00	0.01±0.00	0.01±0.00
α-Cryptoxanthin	0.53±0.01	0.09±0.00	0.08±0.00	0.13±0.00	0.13±0.00	0.36±0.00	0.30±0.00	0.27±0.00
Lutein dilaurate	0.06±0.00	0.03±0.00	0.02±0.00	0.05±0.00	0.03±0.00	0.08±0.00	0.06±0.00	0.03±0.00
Lutein dimyristate	0.10±0.00	0.02±0.00	0.03±0.00	0.05±0.00	0.05±0.00	0.11±0.00	0.06±0.00	0.01±0.00

Table S7 Amino acids changes during black tea processing using different altitude cultivated fresh leaves (mg•g^{-1} DW)

Amino acids	Black tea prepared from high-altitude cultivated fresh leaves				Black tea prepared from low-altitude cultivated fresh leaves			
	FL	BW	BF	BT	FL	BW	BF	BT
Phenylalanine	0.11±0.00	0.98±0.02	0.78±0.01	0.45±0.01	0.04±0.00	0.45±0.01	0.39±0.01	0.12±0.00
Leucine	0.00±0.00	0.57±0.01	0.45±0.01	0.27±0.00	0.00±0.00	0.35±0.01	0.23±0.00	0.02±0.00
Tryptophan	0.44±0.01	0.68±0.01	0.57±0.01	0.42±0.01	0.36±0.01	0.58±0.01	0.45±0.01	0.3±0.00
Valine	0.11±0.00	0.4±0.01	0.35±0.01	0.25±0.00	0.10±0.00	0.37±0.01	0.31±0.01	0.17±0.00
Proline	0.1±0.00	0.43±0.01	0.37±0.01	0.26±0.00	0.10±0.00	0.42±0.01	0.32±0.01	0.16±0.00
Tyrosine	0.6±0.01	0.8±0.01	0.76±0.01	0.67±0.01	0.63±0.01	0.94±0.02	0.89±0.02	0.69±0.01
Alanine	0.29±0.00	0.55±0.01	0.51±0.01	0.38±0.01	0.25±0.00	0.35±0.01	0.32±0.01	0.24±0.00
Threonine	0.35±0.01	0.53±0.01	0.45±0.01	0.30±0.01	0.28±0.00	0.44±0.01	0.34±0.01	0.18±0.00
Glycine	0.13±0.00	0.14±0.00	0.14±0.00	0.13±0.00	0.13±0.00	0.13±0.00	0.14±0.00	0.13±0.00
Glutamine	4.02±0.07	3.32±0.06	2.50±0.04	1.21±0.02	2.47±0.04	1.69±0.03	1.24±0.02	0.68±0.01
Serine	0.88±0.01	1.44±0.02	1.24±0.02	0.76±0.01	0.57±0.01	0.97±0.02	0.68±0.01	0.36±0.01
Glutamic acid	4.03±0.07	3.54±0.06	2.79±0.05	1.57±0.03	3.21±0.05	3.40±0.06	1.38±0.02	0.48±0.01
Asparagine	0.83±0.01	1.37±0.02	1.19±0.02	0.97±0.02	0.77±0.01	1.40±0.02	1.27±0.02	0.88±0.01
aspartic acid	1.70±0.03	1.30±0.02	1.21±0.02	1.06±0.02	1.99±0.03	1.53±0.03	1.22±0.02	0.97±0.02
Histidine	0.39±0.01	0.38±0.01	0.34±0.01	0.33±0.01	0.36±0.01	0.42±0.01	0.33±0.01	0.32±0.01
Arginine	2.57±0.04	2.06±0.03	1.39±0.02	0.65±0.01	1.44±0.02	0.88±0.01	0.65±0.01	0.11±0.00
Lysine	0.11±0.00	0.25±0.00	0.18±0.00	0.12±0.00	0.10±0.00	0.20±0.00	0.13±0.00	0.08±0.00
isoleucine	0.01±0.00	0.03±0.00	0.02±0.00	0.01±0.00	0.01±0.00	0.02±0.00	0.01±0.00	0.01±0.00
Theanine	25.96±0.44	23.67±0.4	20.89±0.35	13.58±0.23	19.62±0.33	15.45±0.26	13.17±0.22	5.82±0.10
